# Bolstering Community Cooperation in Ebola Resurgence Protocols: Combining Field Blood Draw and Point-of-Care Diagnosis

**DOI:** 10.1371/journal.pmed.1002227

**Published:** 2017-01-24

**Authors:** Mosoka P. Fallah, Laura A. Skrip, Philomena Raftery, Miata Kullie, Watta Borbor, A. Scott Laney, David J. Blackley, Athalia Christie, Emily Kainne Dokubo, Terrence Q. Lo, Stewart Coulter, April Baller, Benjamin T. Vonhm, Philip Bemah, Sowillie Lomax, Adolphus Yeiah, Yatta Wapoe-Sackie, Jennifer Mann, Peter Clement, Gloria Davies-Wayne, Esther Hamblion, Caitlin Wolfe, Desmond Williams, Alex Gasasira, Francis Kateh, Tolbert G. Nyenswah, Alison P. Galvani

**Affiliations:** 1 Ministry of Health, Monrovia, Liberia; 2 Community-Based Initiative, United Nations Development Programme, Ministry of Health, Monrovia, Liberia; 3 A.M. Dogliotti College of Medicine, University of Liberia, Monrovia, Liberia; 4 National Institute of Allergy and Infectious Diseases, PREVAIL-III Study, Monrovia, Liberia; 5 Center for Infectious Disease Modeling and Analysis (CIDMA), Yale School of Public Health, New Haven, Connecticut, United States of America; 6 World Health Organization, Monrovia, Liberia; 7 Centers for Disease Control and Prevention, Monrovia, Liberia

## Abstract

Alison Galvani and colleagues describe a community-based protocol to improve cooperation with Ebola testing as well as contact tracing, quarantining, and treatment.

**Summary Points**

- Community resistance arising from fear, stigma, and lingering trauma following the devastating Ebola outbreak has posed challenges for surveillance in Liberia.
- During localized resurgences of Ebola since May 2015, a protocol of field blood draw by trained personnel combined with PCR-based GeneXpert diagnosis was developed as an option for individuals suspected of having Ebola who refused admission to Ebola treatment units (ETUs), where diagnosis is typically conducted.
- This new protocol bolstered community cooperation, accelerated diagnosis, and facilitated rapid isolation of patients with Ebola, which is in turn fundamental to curtailing household transmission and improving prognosis.
- Potential limitations of the protocol include the requirement of deployable human resources with necessary laboratory expertise and proximity to diagnostic facilities. A further consideration is the trade-off between the occupational risk of conducting blood draw in the field and the transmission risk to individuals suspected of having Ebola awaiting results in the ETU.

## Background

On May 9, 2015, the World Health Organization (WHO) declared Liberia to be free of known Ebola virus transmission following a 14-month epidemic that resulted in nearly 4,800 deaths within Liberia alone [1]. Since this declaration, three Ebola resurgences have occurred in Liberia (June 2015, November 2015, March 2016), and others have occurred in Sierra Leone (January 2016) and Guinea (March 2016). Whereas outbreaks of Ebola have been characterized by expansive transmission chains that disseminated over wide geographical scale, these resurgences were successfully contained as localized incidents in areas with recent history of widespread transmission. The risk of Ebola resurgences has been associated with increasing evidence of viral persistence in semen, breast milk, ocular fluid, and immunologically protected tissues during convalescence, with documented cases of sexual transmission from survivors [2–6]. This viral persistence combined with the sheer number (over 10,000) of Ebola survivors currently in West Africa poses a continued threat [7].

Proactive surveillance efforts are fundamental to ensuring that resurgences do not ignite uncontrolled outbreaks. Efforts in Liberia include (1) swabbing and testing of bodies upon death by any cause and (2) investigating patients who present at hospitals or who are identified in communities with Ebola virus disease (EVD)-like symptoms, as prescribed by the WHO Phase III surveillance strategy [8]. Alert cases of a potential recurrence are assessed according to the standard viral hemorrhagic fever suspect case definition (i.e., onset of fever and no response to usual causes of fever in the area) and at least one of the following signs: bloody diarrhea, bleeding from the gums, purpura, bloody urine, or clinical suspicion of EVD [8]. Individuals suspected of having Ebola are admitted to an Ebola treatment unit (ETU), where they undergo two blood draws 48 hours apart. If both tests are negative and once any clinical symptoms have resolved, the individuals are discharged and are observed for 21 days. The postdischarge observation period is in part due to the possibility of EVD exposure while in the ETU [9]. Nosocomial transmission has motivated the consideration of alternative strategies for diagnosing individuals suspected of having Ebola [10,11].

Despite national efforts to develop prevention and preparedness strategies, however, the trauma associated with EVD has thwarted Liberia’s surveillance protocol. In particular, individuals suspected of having Ebola are reluctant to be transported by ambulance to an ETU for diagnosis, largely because of fear of stigma [12–14]. In some cases, individuals suspected of having Ebola hid from response teams or even threatened violence. Such evasion of response efforts could exacerbate disease spread [15–17]. In our response efforts to the Liberian resurgences, the availability of rapid point-of-care diagnostic technology and previous evidence for effective use of home-based and community-based diagnostic and treatment efforts [18,19] helped to overcome logistical and social barriers and provided support for an alternative strategy. This strategy drew upon experiences of response efforts, namely the Community-Based Initiative (CBI), a community-driven approach that engaged affected families and local leaders [20].

Here we propose a diagnostic protocol based on field blood draw and rapid GeneXpert assay that offers individuals suspected of having EVD the option of remaining in their communities during the diagnostic process. We present case studies on the use of field diagnosis from the June 2015 resurgence in Margibi County and the November 2015 and April 2016 resurgences in Montserrado County to underscore the importance of sensitively addressing the concerns that hinder cooperation with surveillance and diagnostic protocols.

## Challenges to Effective and Sustainable Ebola Surveillance

After 42 days of no known EVD transmission, Liberia shifted to a 90-day period of heightened surveillance in May 2015. During this period, the screening criteria for suspected cases of Ebola included febrile illness that did not respond to treatment, or sequelae clinically consistent with Ebola [8]. Challenges (Box 1) to ETU-based diagnosis of individuals suspected of having EVD quickly became apparent. In June 2015, a man was reported to be vomiting by the community-based active case finders (ACFs) responsible for surveillance. An ambulance team was called to investigate and determine if he met the suspected case definition that would warrant ETU admission. Since the man was visiting from Guinea, where there was ongoing Ebola virus transmission, and since he was presenting with symptoms consistent with EVD, the team recommended that he be transported to the ETU for diagnosis. The individual suspected of having Ebola refused ETU admission, and his family threatened the ambulance team with violence. After the ambulance departed, the man fled with his family.

**Box 1. Challenges for EVD Surveillance and Response in Post-outbreak Liberia**

1. In a setting that experiences myriad diseases with overlapping symptoms, Liberia’s surveillance team must be judicious, focusing on cases that warrant strong suspicion of EVD.
2. Fears that linger from the trauma and stigma of the 2014–2015 outbreak must be allayed, and individuals suspected of having Ebola convinced to undergo diagnosis.
3. In the event of new emergence or resurgence, it is imperative to minimize exposure to Ebola among both response staff and community members. Appropriate infection prevention and control measures should be integrated into cultural practices as well as health care protocols and standards. The capacity to implement these protocols requires adequate response infrastructure for isolating individuals with Ebola and averting nosocomial transmission.

In June 2015, a man was wandering the streets of Monrovia and vomiting blood. Recognizing that his symptoms were consistent with the criteria for suspicion of EVD, local ACFs called an ambulance to transport him to an ETU. When the ambulance arrived, the man fled. Further investigation in the community led to the conclusion that his symptoms were due to tuberculosis. If the man had been found to meet the suspected case definition and had been taken to the ETU, he would have not only undergone two tests 48 hours apart for Ebola virus prior to discharge but would have also been subjected to 21 days of monitoring in case he had been exposed to the virus during his ETU admission. Our response team realized the need for an alternative surveillance approach that would be sensitive to communities’ fears and accelerate diagnosis.

## Technological Advances to Accelerate EVD Diagnosis

Traditionally, diagnosis of EVD involves real-time reverse transcription-PCR (RT-PCR) assays, which require 2 to 6 hours once a sample is received [21–24]. In 2015, the United States Food and Drug Administration and the WHO authorized emergency use of the Cepheid GeneXpert Ebola assay, a point-of-care diagnostic test that uses whole blood versus serum or plasma and accelerates results [26–28]. The sensitivity and specificity of the assay are comparable to those of the standard real-time RT-PCR, with enhanced sensitivity for the nucleoprotein target [21,26]. Specifically, compared to the benchmark Trombley real-time RT-PCR (rRT-PCR) assay for detection of the nucleoprotein of the Ebola virus [29], sensitivity and specificity on whole blood samples were found to be 100% (95% CI: 84.6%–100%) and 95.8% (95% CI: 91.8%–98.2%), respectively, for GeneXpert [26]. The feasibility of implementing the technology, with its automated and closed-cartridge system for ease and safety, has been evaluated in an ETU in Guinea [27] and a field biocontainment laboratory in Sierra Leone [26].

## Community Cooperation to Ebola Response Efforts Garnered through Field Blood Draw

In June 2015, positive samples from postmortem buccal swab and cardiac puncture prompted activation of an EVD response in Liberia’s Margibi County [30]. When the response team arrived to investigate, contacts of the alert case refused referral to an ETU for diagnosis. Despite the positive EVD result from the body of the deceased, his contacts attributed his death to malaria. With the response team serving as facilitators, a subsequent discussion and negotiation then took place among the community leaders, key stakeholders, and the affected families who shared households with the 51 high-risk contacts. It was agreed that the high-risk contacts would cooperate with home-based blood draw and then admission to ETU in the event of positive diagnosis.

By addressing the concerns of community members, the proposed approach was expected to improve cooperation with Ebola testing as well as contact tracing, quarantining, and treatment. The use of a readily deployable team of trained personnel reduced the risk of individuals suspected of having Ebola evading response efforts and/or seeking care from primary health care settings unequipped for Ebola prevention and control, thereby also reducing the occupational risk within primary health care settings that was so problematic during Ebola outbreaks. Community awareness and acceptance of field diagnosis could also reduce reliance on traditional medicine, which exacerbated transmission in a number of cases.

While personal protective equipment (PPE) is the gold standard barrier precaution for reducing occupational risk of Ebola transmission, there was concern about the stigma associated with the arrival of an Ebola team in full PPE. The new protocol (Box 2) was designed to ensure that biosafety measures were implemented while allowing the team to arrive in normal clothing and then discreetly don basic PPE before engaging with the individual suspected of having Ebola. The protocol reflected response experiences in the Margibi resurgence as well as expert review by Liberia’s National Ebola response team, the WHO, and the Centers for Disease Control and Prevention (CDC).

**Box 2. Protocol for Field Diagnosis in Communities with High-Risk Contacts of a Confirmed EVD Case**

1. Willingness of individuals suspected of having Ebola and their contacts to be admitted to an ETU for testing and observation is assessed.
2. If individuals are resistant to ETU admission, the field blood draw approach is presented as an alternative.
3. A phlebotomist with basic personal protective equipment (PPE) is on standby to perform field blood draws. Basic PPE included rain boots, gloves, a surgical gown, and head gear. Biohazard bags are available for discarding blood draw equipment. Full PPE could be used at the discretion of the blood draw team.
4. To ensure privacy of the individual being tested as well as avoiding the need for the phlebotomist and an assistant to enter a potentially contaminated home, the phlebotomist performs the blood draw just outside the home of the individual suspected of having Ebola or in the back of the team’s canopied truck.
5. A laboratory assistant, also dressed in basic PPE, holds the biohazard bag, and another laboratory assistant sprays used blood draw equipment with chlorine solution before placing in bag.
6. A phone call is made to the Ministry of Health and WHO Laboratory Coordination team to alert the nearest facility equipped with GeneXpert to be prepared for sample delivery.
7. Pending the test results (typically received within 6 hours), the individual suspected of having Ebola is instructed to self-isolate in a room apart from other household members. To ensure the isolation of the individual suspected of having Ebola and to monitor any change in symptoms, the field team visits, but does not enter, the household.
8. Results are transmitted via short message service (SMS), email, and phone to essential personnel for immediate ETU admission of individuals who test positive for EBV or to assuage concerns if the test result is negative.
9. If the test result is negative, a second blood draw and confirmatory diagnosis is conducted 48 hours later. During the intervening period, response workers monitor the household at least twice daily.

Receptiveness to this rapid diagnostic approach facilitated control of the Margibi resurgence. During this first resurgence, a total of 19 field blood tests were conducted in a community with a population size of 3,498 members; five community members tested positive and were admitted to the ETU. In this resurgence, the time between field blood draw and receipt of diagnosis ranged from 2 to 4 hours. All of the five individuals with confirmed Ebola survived, due at least in part to their swift identification, compliance with the rapid diagnostic protocol, and timely clinical management in the ETU. Also of great importance, by engaging high-risk contacts in testing and isolation, the field protocol likely reduced the risk of household transmission, which rises substantially over the course of the symptomatic period. In particular, rapid isolation, specifically within 4 days of symptom onset, has been shown to be critical for reducing transmission [31].

## Implementation of Field Diagnosis in the Second and Third Ebola Resurgences

Following the success of the field diagnosis protocol in the first Liberian resurgence, it was again implemented in response to a November 2015 EVD cluster in a suburb of Monrovia. The alert case of this second resurgence was reported to the team by staff at a hospital where he sought care. The next morning, an extensive list was generated of over 165 contacts, who were visited in their homes twice daily by the response team to assess their temperature and the onset of any symptoms. Within 48 hours from the initiation of contact tracing, a contact became febrile but refused to be admitted to the ETU for diagnosis. Instead, field blood draw was conducted, and negative results were returned within 5 hours. As per the protocol, blood draw was repeated 48 hours later, and the results were negative again. A total of eight people underwent field blood draws, for which results were returned within 4 to 6 hours. Though the eight contacts had presented with EVD-like symptoms, the field blood draw results returned negative for all of them, eliminating the need for any ETU admission beyond the original case.

The third resurgence of EVD in Liberia occurred in March 2016. At that point, Ebola surveillance and response, including field blood draw, was occurring alongside a clinical trial of the recombinant vesicular stomatitis virus-based candidate vaccine expressing the Zaire Ebola virus glycoprotein (rVSV-ZEBOV). As part of the double ring vaccination trial design, the vaccine was administered to high-risk contacts of alert cases (i.e., primary contacts) as well as high-risk contacts of these contacts (i.e., secondary contacts) [32]. As the vaccine was under clinical trial investigation and there was still a risk for developing EVD even among vaccinated contacts, the field blood draw team remained integral to response efforts. The option for field diagnosis improved vaccine acceptance by helping to overcome a concern that vaccine side effects, namely fever [33], could lead to ETU admission. The field blood draw team took samples for GeneXpert testing of viral nucleoprotein and glycoprotein from any primary contacts who became symptomatic. If any such samples tested positive, additional lab assessment would then be conducted to distinguish reactivity attributable to the vaccine versus actual viremia. In this third resurgence, two vaccinated primary contacts developed fever and had blood drawn. Their GeneXpert results were negative, suggesting that symptoms were mild vaccine side effects.

## Potential Drawbacks and Future Recommendations for Field Blood Draw

Point-of-care assays combined with field blood draw have the potential to accelerate diagnoses in emergency settings, where time is critical both for reducing Ebola transmission and improving prognosis. The feasibility of the point-of-care assay in a wider range of settings than is possible for more traditional methods and the rapidity of diagnostic results offer additional flexibility for responding to outbreaks of highly infectious diseases. This flexibility was fundamental to fostering cooperation with community members in EVD response efforts when ETU admission was associated with trauma, stigma, and potential nosocomial infection. Counter to initial fears, none of the phlebotomists, assistants, or household members became infected.

Despite the successful implementation of the protocol in the resurgences to date, several potential drawbacks exist. As time from the initial epidemic elapses, declining adherence to prevention recommendations has been observed, exemplified by increased consumption of bushmeat [34,35]. Likewise, cooperation with field diagnosis may erode. Another potential drawback is dependence on geographic proximity and accessibility to a GeneXpert instrument. If the affected community is in a remote location or roads are impassable, sample transport from the field to a GeneXpert instrument, and thus diagnosis, could be delayed. Furthermore, lack of adequately trained personnel to safely conduct the protocol could become an obstacle. Sustainable expansion of phlebotomy and laboratory capacity should involve training on safety measures related to infection prevention and control to determine and maintain standards that are acceptable to authorities, response workers, and community members. Thus, possible differences in opinion regarding the use of basic versus full PPE in field blood draw, for instance, could be addressed ahead of new resurgences.

Given the record of thus far overcoming social, geographic, and human capacity challenges, continued scale-up of the protocol will be an important next step in epidemic preparedness. An implementation plan has been drafted to expand GeneXpert capacity throughout Liberia and integrate testing for other infectious diseases, such as tuberculosis (TB), in order to ensure sustainable and efficient use of the instruments. In response to and following the EVD crisis, 14 GeneXpert instruments have been installed at six laboratories throughout Liberia: three facilities (at John F. Kennedy [JFK] Hospital, Redemption Hospital, and Eternal Love Winning Africa [ELWA] ETU) in Montserrado and the National Reference Lab in Margibi, as well as regional laboratories in Bong and Tappita.

Other non-PCR, point-of-use diagnostics could be used to supplement RT-PCR approaches, such as in settings where GeneXpert technology is unavailable or infeasible [36]. The Defence Science and Technology Laboratory rapid diagnostic test, for example, has been shown to achieve 100% sensitivity and 92% specificity relative to PCR methods and only requires a blood volume attainable from a finger stick [36]. In accordance with WHO guidelines [8], non-PCR rapid diagnostic tests are encouraged when the time to obtain results by PCR would exceed 72 hours, although reactive results from a non-PCR test warrant PCR confirmation [8]. In parallel, the possibility of scaling back ETU personnel that would be required for the 48-hour monitoring of individuals suspected of having Ebola could be evaluated. This aligns with the anticipated closure of all ETUs and the decentralization of identification, testing, and care [37]. Through ongoing and prospective efforts, Ebola preparedness teams can achieve an improved surveillance approach that balances risk with the contextual sensitivity required to most effectively implement proven outbreak response strategies.

## Abbreviations

ACF: active case finder
CBI: Community-Based Initiative
CDC: Centers for Disease Control and Prevention
ELWA: Eternal Love Winning Africa
ETU: Ebola treatment unit
EVD: Ebola virus disease
JFK: John F. Kennedy
PPE: personal protective equipment
rRT-PCR: real-time reverse transcription-PCR
RT-PCR: reverse transcription-PCR
rVSV-ZEBOV: recombinant vesicular stomatitis virus-based candidate vaccine expressing the Zaire Ebola virus glycoprotein
TB: tuberculosis
WHO: World Health Organization
